# The impact of statin use on short-term and long-term mortality in patients with heart failure

**DOI:** 10.3389/fphar.2024.1397763

**Published:** 2024-09-26

**Authors:** Xiaoxue Zheng, Long Tan, Yu Zhang

**Affiliations:** ^1^ Department of Healthcare, Beijing Hospital, National Center of Gerontology, Institute of Geriatric Medicine, Chinese Academy of Medical Sciences, Beijing, China; ^2^ Health Service Department, Guard Bureau of the General Office of the Central Committee of the Communist Party of China, Beijing, China

**Keywords:** statin, heart failure, all-cause mortality, intensive care unit, MIMIC-IV database

## Abstract

**Background:**

Heart failure (HF) is a complex disorder that has an association with increased morbidity and mortality rates globally. The association of statin use with mortality rate in individuals with HF remains unclear.

**Objectives:**

To examine the association of statin use with the short-term and long-term all-cause mortality rate in critically ill individuals with HF.

**Methods:**

We performed a retrospective cohort analysis based on the Medical Information Mart for Intensive Care (MIMIC)-IV database. The critically ill people with HF were assigned to a statin group and a non-statin group according to whether they had been treated with statin or not during hospitalization. The Kaplan−Meier (KM) method and Cox proportional hazard models were adopted to explore the link between statin administration and the 30-day, 90-day, as well as 1-year mortality rates. To ensure the robustness of the findings, a 1:1 nearest propensity-score matching (PSM) was also performed.

**Results:**

The current research included 11,381 patients for the final analysis, with 7,561 in the statin group and 3,820 in the non-statin group. After multiple confounders were adjusted, we found that the Cox regression models revealed great beneficial effects of statin therapy on the 30-day, 90-day, as well as 1-year mortality rates among critically ill individuals with HF in the fully adjusted model. PSM also achieved consistent results. After PSM, the risk of mortality reduced by 23% for the 30-day mortality (HR = 0.77, 95%CI: 0.68–0.88, *p* < 0.001), 16% for the 90-day mortality rate (HR = 0.84, 95%CI: 0.75–0.93, *p* < 0.001), and 12% for the 1-year mortality rate (HR = 0.88, 95%CI: 0.81–0.97, *p* = 0.007). Patients treated with rosuvastatin had the greatest reduction in mortality rate. The 30-day, 90-day, and 1-year all-cause mortality rates were remarkably lower in patients who were treated with low-dose statins.

**Conclusion:**

Our study unveiled that statin use was related to decreased short-term and long-term all-cause mortality rates in critically ill individuals with HF. Rosuvastatin was associated with the greatest reduction of all-cause mortality rates. Low-dose statins can significantly reduce short-term and long-term mortality, while high-dose statins are not significantly correlated with mortality. However, the results are not conclusive and should be interpreted with caution.

## 1 Introduction

Heart failure (HF), a cardiovascular condition, is growing rapidly in the world. It is a complex disorder related to increased morbidity and mortality rates and poses a great burden to the healthcare system (Ziaeian and Fonarow, 2016). There will likely be a 24% rise in the estimated prevalence of HF in the U.S., implicating more than 8 million people in 2030 (Heidenreich et al., 2013).

Statins, 3-hydroxy-3-methyl glutaryl-coenzyme A reductase (HMG-CoA) reductase inhibitors, have been conclusively demonstrated to lower the risk of major coronary events, stroke, as well as coronary revascularisation (Baigent et al., 2005). However, there is still controversy over whether statin therapy can lower the mortality rate in HF patients. Treatment with statins did not influence the morbidity and mortality in people with chronic HF of any cause in two large-scale placebo-controlled trials of individuals with established HF (Tavazzi et al., 2008; Kjekshus et al., 2007). In contrast, Foody et al. (2006) found that statin therapy had an association with lower long-term mortality in older people with HF. In 2019, Bielecka-Dabrowa et al. (2019) studied the effects of statin treatment in individuals with HF using a meta-analysis, and they found that statin use was linked to lower all-cause mortality rate, cardiovascular mortality rate, as well as cardiovascular hospitalization in HF with reduced ejection fraction (HFrEF) and HF with preserved ejection fraction (HFpEF). The latest meta-analysis that was published in 2023 also reported a decrease in all-cause mortality rate in individuals with HFpEF who received statin therapy (Kaur et al., 2023).

Uncertainty has arisen from studies on the relationship between statin use and all-cause mortality rate in people with HF. The current study was carried out to explore the association of statin use with all-cause mortality rates in critically ill individuals with HF using the Medical Information Mart for Intensive Care (MIMIC)-IV database.

## 2 Materials and methods

### 2.1 Data source

Data in this retrospective cohort study were extracted from the MIMIC-IV database version 2.2 (https://physionet.org/content/mimiciv/2.2/). As a large, single-center, freely and publicly available medical information database, the MIMIC-IV contains comprehensive patient information that is maintained by the Beth Israel Deaconess Medical Center. MIMIC-IV contains over 250,000 emergency department admissions as well as over 60,000 ICU stays from 2008 to 2019. An author of this study has obtained the certificate (No: 58380649) after finishing the relevant training courses to gain access to the database.

### 2.2 Participants

The critically ill patients who were diagnosed with HF based on the ICD-9 and ICD-10 were initially screened. The exclusion criteria were set as follows: 1) age <18 years, 2) age >100 years, 3) not the first hospitalization, and 4) death time earlier than admission time.

### 2.3 Data extraction

Admission data were obtained by using the structured Query Language with PostgreSQL (version 16) from MIMIC-Ⅳ (Wu et al., 2021). We extracted the following variables: 1) Demographic characteristics, such as age, weight, race, gender, and admission type. 2) Comorbidities like hypertension, diabetes, myocardial infarct (MI), atrial flutter (AFL) or atrial fibrillation (AF), peripheral vascular disease (PVD), hyperlipidemia, stroke, and chronic kidney disease (CKD). 3) Scoring systems like the Charlson Comorbidity Index, the Sequential Organ Failure Assessment (SOFA) score, as well as the Acute Physiology Score III (APSIII). 4) Vital signs during the first 24 h of admission to the ICU, such as heart rate (HR), respiratory rate (RR), systolic blood pressure (SBP), diastolic blood pressure (DBP), and oxygen saturation (SpO2). 5) Baseline laboratory parameters during the first 24 h of admission to the ICU, such as white blood cells (WBC), prothrombin time (PT), red blood cells (RBC), platelet, international normalized ratio (INR), red blood cell distribution width (RDW), hematocrit, hemoglobin, sodium, potassium, chloride, glucose, anion gap, partial thromboplastin time (PTT), urea nitrogen, and creatinine. 6) Clinical therapies, including mineralocorticoid receptor antagonist (MRA), digoxin, loop diuretic, angiotensin II receptor blocker (ARB), angiotensin-converting enzyme inhibitor (ACEI), mechanical ventilation, β-blockers, anti-platelet drugs, intra-aortic balloon pump (IABP), continuous renal replacement therapy (CRRT), and vasopressor.

### 2.4 Outcome indicators

The primary endpoint in the current research was the 1-year all-cause mortality rate in people with HF after admission, and the secondary endpoints included the 30-day mortality rate as well as 90-day mortality rate.

### 2.5 Statistical analysis

According to whether statin was used or not, patients were grouped into the non-statin group and the statin group. To minimize biases resulting from missing data, we excluded variables that had over 30% missing data and used multiple imputation to duplicate other data (Cummings, 2013). The mean ± standard-deviation values were used to display continuous variables that were normally distributed, while quartiles were adopted to present continuous variables that were non-normally distributed. Student’s t-test and Wilcoxon rank sum test were employed to evaluate the difference significance between the statin and non-statin groups. Categorical variables were presented as frequencies and percentages, and the chi-square test was adopted to assess differences between the two groups.

We used the Kaplan-Meier (K-M) to estimate the 30-day, 90-day and 1-year survival probabilities of patients from the statin and non-statin groups. With the Cox proportional hazard models, the effect of statin treatment on mortality was assessed using hazard ratios (HRs) and 95% confidence intervals (CIs). Patients from the non-statin group were taken as a reference to establish three Cox proportional-hazards regression models: 1) Model I, unadjusted model; 2) Model II, with age, gender, race, admission type, weight, HR, SBP, DBP, RR, SpO2, hypertension, diabetes, myocardial infarction, PVD, stroke, hyperlipidemia, AFL or AF and CKD included; 3) Model III, the Model II plus SOFA, Charlson comorbidity index, APSIII, WBC, RBC, platelet, hemoglobin, RDW, hematocrit, sodium, potassium, chloride, glucose, anion gap, PT, PTT, INR, urea nitrogen, creatinine, CRRT, ventilation, IABP, vasoactive drugs, MRA, ACEI, ARB, β-blocker, digoxin, loop diuretic, and antiplatelet drugs. The goodness-of-fit of models was evaluated using the Akaike information criterion (AIC) and Bayesian information criterion (BIC) (Wu et al., 2023). The association of the types and doses of statins with mortality rate was also evaluated. We performed subgroup analysis to investigate whether the demographic features and comorbidities affect the association of statin administration with mortality rate. In each type and dose of statins, Cox proportional-hazards regression analysis in the fully adjusted model (model III) was performed.

To lower the imbalance in the baseline covariates between groups, an algorithm of 1:1 greedy matching was applied in the propensity-score matching (PSM) method. In the PSM model, one-to-one nearest neighbor matching with a caliper width of 0.05 was employed in the present study. Two-sided p-values below 0.05 indicated that the difference was statistically significant. R (version 4.2.3) was adopted for all the statistical analyses.

## 3 Results

### 3.1 Baseline features

**FIGURE 1:**
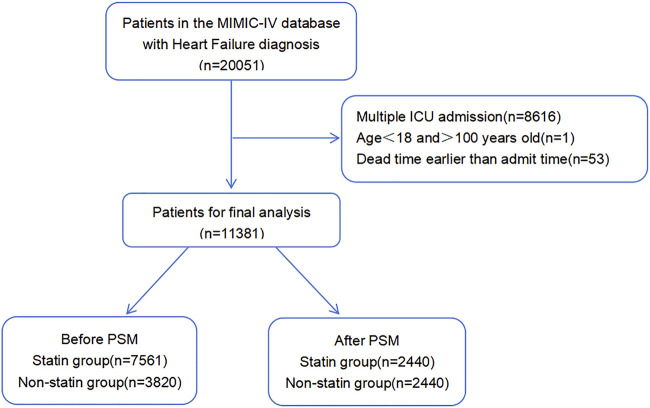
Selection of study population from MIMIC-IV database.

As shown in Figure 1, data on 20,051 patients with HF were extracted from the MIMIC-IV database. After removing patients aged <18 and >100 years, those who had previously been hospitalized, and patients whose death time was earlier than the admission time, we included 11,381 patients in the final analysis.

Before PSM, 7,561 patients who received statins were assigned to the statin group and 3,820 patients who received no statins were grouped to the non-statin group. The median age of the population in the original cohort was 73.86 ± 13.43 years old (IQR: 18–100), among whom 6,252 (54.93%) were males and 7,912 (69.52%) were white people. Differences in the baseline characteristics between the statin and non-statin groups can be seen in Table 1.

**TABLE 1:** Baseline characteristics between groups before and after PSM.

Variable	Before PSM					After PSM				
	Total (n = 11,381)	Non-statin (n = 3,820)	Statin (n = 7,561)	*P* value	SMD	Total (n = 4,880)	Non-statin (n = 2,440)	Statin (n = 2,440)	*P* value	SMD
Demographics										
Age (years)	73.86 ± 13.43	72.10 ± 16.09	74.74 ± 11.76	<.001	0.225	74.13 ± 13.55	73.89 ± 14.63	74.37 ± 12.37	0.215	0.039
Weight (kg)	83.83 ± 25.62	82.29 ± 28.42	84.61 ± 24.04	<.001	0.097	82.76 ± 26.32	83.33 ± 28.01	82.19 ± 24.50	0.130	−0.047
Gender				<0.001					0.647	
Female	5129 (45.07)	1957 (51.23)	3172 (41.95)		−0.188	2411 (49.41)	1198 (49.10)	1213 (49.71)		0.012
Male	6252 (54.93)	1863 (48.77)	4389 (58.05)		0.188	2469 (50.59)	1242 (50.90)	1227 (50.29)		−0.012
Race				<0.01					0.945	
Black	1111 (9.76)	427 (11.18)	684 (9.05)		−0.074	536 (10.98)	270 (11.07)	266 (10.90)		−0.005
Other	981 (8.62)	358 (9.37)	623 (8.24)		−0.041	409 (8.38)	211 (8.65)	198 (8.11)		−0.020
Unknown	1377 (12.1)	509 (13.32)	868 (11.48)		−0.058	620 (12.7)	308 (12.62)	312 (12.79)		0.005
White	7912 (69.52)	2526 (66.13)	5386 (71.23)		0.113	3315 (67.93)	1651 (67.66)	1664 (68.20)		0.011
Admission type				<0.01					0.930	
Elective	539 (4.74)	136 (3.56)	403 (5.33)		0.079	225 (4.61)	108 (4.43)	117 (4.80)		0.017
Emergency	5,969 (52.45)	2,237 (58.56)	3,732 (49.36)		−0.184	2,742 (56.19)	1,383 (56.68)	1359 (55.70)		−0.020
Observation	1,296 (11.39)	406 (10.63)	890 (11.77)		0.035	507 (10.39)	255 (10.45)	252 (10.33)		−0.004
Surgical same day	689 (6.05)	185 (4.84)	504 (6.67)		0.073	287 (5.88)	146 (5.98)	141 (5.78)		−0.009
Urgent	2,888 (25.38)	856 (22.41)	2,032 (26.87)		0.101	1,119 (22.93)	548 (22.46)	571 (23.40)		0.022
Vital signs										
Heart Rate(beats/min)	84.07 ± 15.74	86.99 ± 17.11	82.60 ± 14.79	<0.001	−0.297	84.81 ± 16.23	84.98 ± 16.33	84.65 ± 16.13	0.481	−0.020
SBP(mmHg)	115.81 ± 17.85	114.80 ± 18.29	116.33 ± 17.61	<0.001	0.087	116.08 ± 18.03	115.92 ± 18.19	116.24 ± 17.87	0.534	0.018
DBP(mmHg)	62.08 ± 11.67	62.86 ± 12.13	61.69 ± 11.41	<0.001	−0.103	62.46 ± 11.84	62.45 ± 11.94	62.48 ± 11.74	0.920	0.003
Respiratory Rate(beats/min)	19.82 ± 3.75	20.30 ± 4.05	19.57 ± 3.56	<0.001	−0.205	19.94 ± 3.78	19.91 ± 3.82	19.97 ± 3.75	0.597	0.015
SpO_2_(%)	96.51 ± 2.43	96.28 ± 2.77	96.63 ± 2.23	<0.001	0.155	96.45 ± 2.46	96.46 ± 2.38	96.45 ± 2.53	0.947	−0.002
Scoring systems										
SOFA	5.13 ± 3.35	5.56 ± 3.66	4.92 ± 3.16	<0.001	−0.201	5.21 ± 3.39	5.20 ± 3.40	5.22 ± 3.37	0.876	0.004
Charlson comorbidity index	6.88 ± 2.56	6.40 ± 2.75	7.12 ± 2.43	<0.001	0.299	6.72 ± 2.58	6.68 ± 2.71	6.75 ± 2.44	0.301	0.031
APSIII	47.71 ± 19.41	51.04 ± 21.76	46.02 ± 17.88	<0.001	−0.281	48.79 ± 19.86	48.62 ± 19.94	48.96 ± 19.77	0.553	0.017
Laboratory measurements										
WBC (10^9^/L)	12.29 ± 7.93	12.43 ± 8.38	12.22 ± 7.70	0.182	−0.028	12.32 ± 8.21	12.39 ± 8.61	12.25 ± 7.79	0.549	−0.018
RBC (10^12^/L)	3.54 ± 0.69	3.55 ± 0.73	3.54 ± 0.68	0.383	−0.018	3.55 ± 0.70	3.55 ± 0.69	3.56 ± 0.70	0.527	0.018
Platelet (10^9^/L)	203.86 ± 94.64	202.53 ± 103.86	204.54 ± 89.62	0.308	0.022	204.87 ± 97.38	203.84 ± 98.75	205.91 ± 96.00	0.458	0.022
Hemoglobin (g/dL)	10.47 ± 1.98	10.51 ± 2.07	10.45 ± 1.93	0.146	−0.030	10.50 ± 1.99	10.50 ± 1.98	10.49 ± 2.01	0.855	−0.005
RDW (%)	15.49 ± 2.24	15.80 ± 2.41	15.34 ± 2.13	<0.001	−0.215	15.65 ± 2.27	15.63 ± 2.30	15.67 ± 2.23	0.562	0.017
Hematocrit (%)	32.13 ± 5.87	32.35 ± 6.28	32.01 ± 5.65	0.005	−0.060	32.27 ± 5.93	32.29 ± 5.94	32.25 ± 5.93	0.844	−0.006
Sodium (mmol/L)	138.29 ± 4.66	138.34 ± 5.12	138.26 ± 4.41	0.458	−0.016	138.38 ± 4.72	138.26 ± 4.77	138.50 ± 4.67	0.085	0.050
Potassium (mmol/L)	4.30 ± 0.58	4.27 ± 0.61	4.31 ± 0.56	0.002	0.065	4.27 ± 0.59	4.28 ± 0.59	4.27 ± 0.58	0.573	−0.016
Chloride (mmol/L)	102.80 ± 6.15	102.63 ± 6.68	102.88 ± 5.86	0.052	0.042	102.76 ± 6.25	102.62 ± 6.33	102.90 ± 6.16	0.112	0.046
Glucose (mg/dL)	144.16 ± 56.61	138.45 ± 55.31	147.04 ± 57.04	<0.001	0.151	140.35 ± 53.52	140.89 ± 56.32	139.81 ± 50.57	0.484	−0.021
Anion gap (mEq/L)	14.89 ± 3.77	15.14 ± 4.11	14.76 ± 3.58	<0.001	−0.108	14.96 ± 3.91	14.97 ± 3.96	14.95 ± 3.86	0.882	−0.004
PT (seconds)	17.48 ± 9.45	18.45 ± 10.91	16.99 ± 8.58	<0.001	−0.170	18.18 ± 10.41	18.24 ± 10.64	18.13 ± 10.17	0.721	−0.010
PTT (seconds)	41.96 ± 22.03	40.31 ± 20.98	42.79 ± 22.50	<0.001	0.110	40.39 ± 21.13	40.26 ± 21.08	40.53 ± 21.18	0.662	0.012
INR	1.61 ± 0.95	1.71 ± 1.11	1.57 ± 0.85	<0.001	−0.169	1.68 ± 1.06	1.69 ± 1.07	1.68 ± 1.04	0.791	−0.008
Urea nitrogen (mg/dL)	33.87 ± 23.73	34.55 ± 25.10	33.53 ± 23.00	0.035	−0.044	34.37 ± 24.20	34.38 ± 24.17	34.36 ± 24.23	0.975	−0.001
Creatinine (mg/dL)	1.72 ± 1.57	1.72 ± 1.67	1.72 ± 1.52	0.953	0.001	1.75 ± 1.68	1.71 ± 1.60	1.79 ± 1.75	0.082	0.048
Treatment during hospitalization										
CRRT	532 (4.67)	202 (5.29)	330 (4.36)	0.028	−0.045	236 (4.84)	123 (5.04)	113 (4.63)	0.505	−0.020
Noninvasive Mechanical Ventilation	581 (5.1)	188 (4.92)	393 (5.20)	0.527	0.012	262 (5.37)	120 (4.92)	142 (5.82)	0.162	0.039
Invasive Mechanical Ventilation	4,181 (36.74)	1,393 (36.47)	2,788 (36.87)	0.670	0.008	1812 (37.13)	893 (36.60)	919 (37.66)	0.441	0.022
IABP	361 (3.17)	51 (1.34)	310 (4.10)	<0.001	0.139	85 (1.74)	42 (1.72)	43 (1.76)	0.913	0.003
Vasoactive	3,851 (33.84)	1,318 (34.50)	2,533 (33.50)	0.286	−0.021	1,640 (33.61)	814 (33.36)	826 (33.85)	0.716	0.010
MRA	1,070 (9.4)	338 (8.85)	732 (9.68)	0.150	0.028	421 (8.63)	192 (7.87)	229 (9.39)	0.059	0.052
ACEI	3,938 (34.6)	962 (25.18)	2,976 (39.36)	<0.001	0.290	1,470 (30.12)	730 (29.92)	740 (30.33)	0.755	0.009
ARB	1,305 (11.47)	277 (7.25)	1,028 (13.60)	<0.001	0.185	426 (8.73)	219 (8.98)	207 (8.48)	0.543	−0.018
βblocker	8,266 (72.63)	2,421 (63.38)	5,845 (77.30)	<0.001	0.333	3,382 (69.3)	1,699 (69.63)	1,683 (68.98)	0.619	−0.014
Digoxin	1,267 (11.13)	488 (12.77)	779 (10.30)	<0.001	−0.081	600 (12.3)	292 (11.97)	308 (12.62)	0.486	0.020
Antiplatelet	8,183 (71.9)	1,771 (46.36)	6,412 (84.80)	<0.001	1.071	3,163 (64.82)	1,599 (65.53)	1,564 (64.10)	0.076	−0.030
Loop diuretic	9,392 (82.52)	2,987 (78.19)	6,405 (84.71)	<0.001	0.181	3,982 (81.6)	1,967 (80.61)	2,015 (82.58)	0.294	0.052
Comorbidities										
Hypertension	3,532 (31.03)	1,156 (30.26)	2,376 (31.42)	0.206	0.025	1,541 (31.58)	783 (32.09)	758 (31.07)	0.441	−0.022
Diabetes	4,653 (40.88)	1,099 (28.77)	3,554 (47.00)	<0.001	0.365	1,735 (35.55)	881 (36.11)	854 (35.00)	0.419	−0.023
Myocardial infarction	2,930 (25.74)	340 (8.90)	2,590 (34.25)	<0.001	0.534	630 (12.91)	313 (12.83)	317 (12.99)	0.864	0.005
PVD	1,766 (15.52)	404 (10.58)	1362 (18.01)	<0.001	0.194	656 (13.44)	320 (13.11)	336 (13.77)	0.502	0.019
Stroke	1,148 (10.09)	321 (8.40)	827 (10.94)	<0.001	0.081	457 (9.36)	228 (9.34)	229 (9.39)	0.961	0.001
Hyperlipidemia	5,911 (51.94)	1,103 (28.87)	4,808 (63.59)	<0.001	0.721	2,032 (41.64)	995 (40.78)	1,037 (42.50)	0.223	0.035
AFL/AF	5,887 (51.73)	2,005 (52.49)	3,882 (51.34)	0.249	−0.023	2,683 (54.98)	1366 (55.98)	1,317 (53.98)	0.159	−0.040
CKD	4,346 (38.19)	1,223 (32.02)	3,123 (41.30)	<0.001	0.189	1,800 (36.89)	891 (36.52)	909 (37.25)	0.593	0.015

SBP, systolic blood pressure; DBP, diastolic blood pressure; SpO_2_, xygen saturation; SOFA, sequential organ failure assessment; APSIII, Acute Physiology Score III; WBC, white blood cells; RBC, red blood cells; RDW, red blood cell distribution width; PT, prothrombin time; PTT, partial thromboplastin time; INR, international normalized ratio; CRRT, continuous renal replacement therapy; IABP, intra-aortic balloon pump; MRA, mineralocorticoid receptor antagonist; ACEI, angiotensin-converting enzyme inhibitor; ARB, angiotensin II, receptor blocker; PVD, peripheral vascular disease; AFL, atrial flutter; AF, atrial fibrillation; CKD, chronic kidney disease.

On the whole, patients from both groups had unbalanced baseline characteristics for most variables. After PSM, 4,880 patients including 2,440 statin users and 2,440 non-statin users were included in the final analysis, and both groups had well-balanced baseline profiles, with P > 0.05 for all variables, as shown in Table 1.

### 3.2 Survival analysis

**FIGURE 2:**
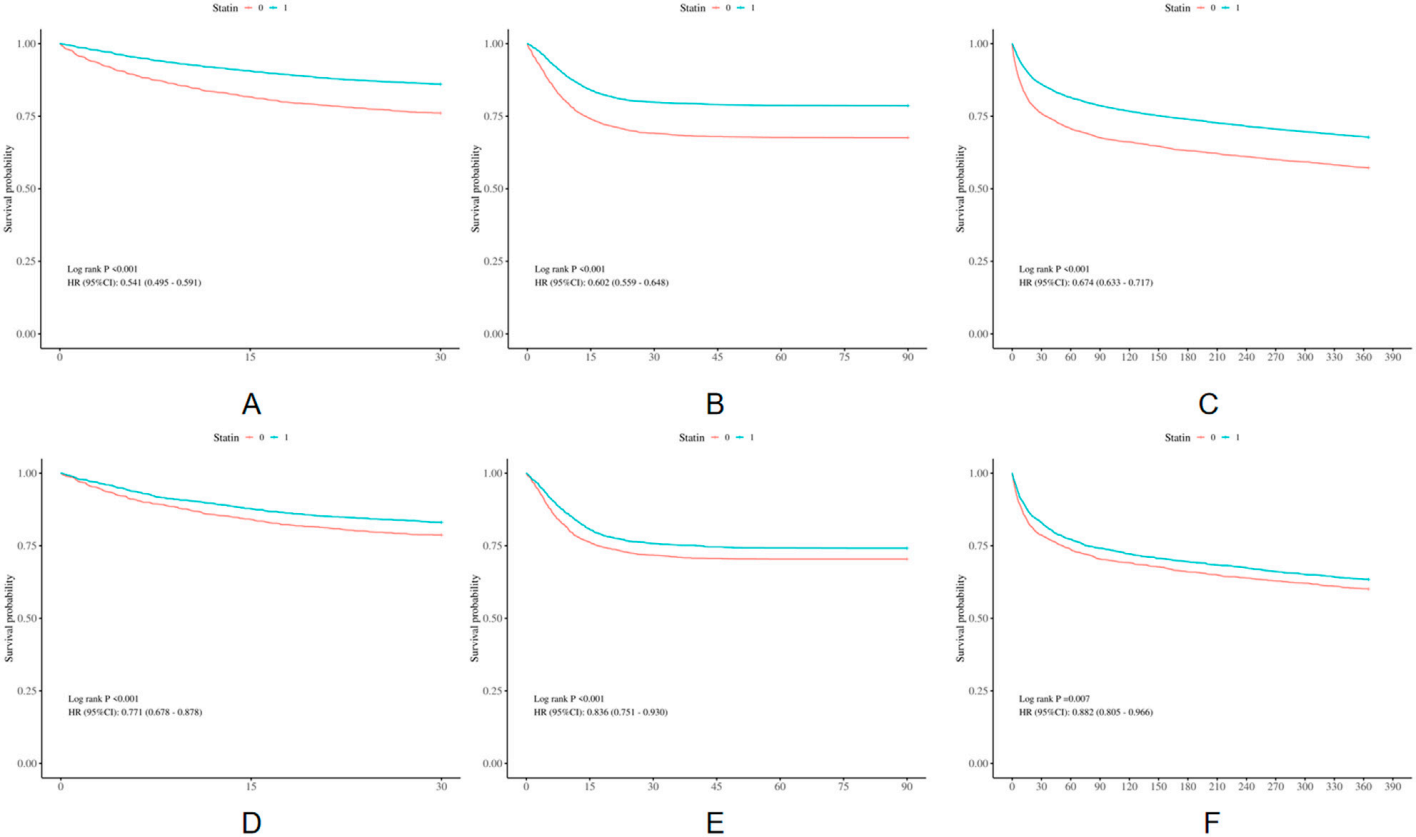
Kaplan-Meier survival curves of the non-statin group and statin group. **(A)** 30-day mortality before PSM; **(B)** 90-day mortality before PSM; **(C)** 1-year mortality before PSM; **(D)** 30-day mortality after PSM; **(E)** 90-day mortality after PSM; **(F)** 1-year mortality after PSM.

The differences in all-cause mortality during different follow-up periods (30 days, 90 days, and 1 year) were assessed between the two groups. For the population in the original cohorts, the statin group had a lower 30-day, 90-day and 1-year mortality in comparison with the non-statin group (*p* < 0.001), as indicated in Figure 2. After PSM, KM survival curves showed consistent results with that of the population in the original cohorts (Figure 2). The results are displayed in Supplementary Table S1.

### 3.3 Association between statin use and all-cause mortality rate

Using Cox proportional hazard models, we further examined the association of statin administration with prognosis, as shown in Table 2. According to a crude model of univariate Cox regression analysis, statin use was remarkably related to a 46%, 40%, 33% reduction in the risk of the 30-day (HR = 0.54, 95%CI:0.49–0.59, *P* < 0.001), 90-day (HR = 0.60, 95%CI:0.56–0.65, *P* < 0.001) and 1-year (HR = 0.67, 95%CI: 0.63–0.72, *P* < 0.001) mortality rates, respectively, in the population in the original cohorts. After adjusting for multiple confounders, as shown in Table 2, in comparison with the non-statin group, the mortality rate of the statin group reduced by 18% within the 30-day follow-up period (HR = 0.82, 95%CI: 0.74–0.92, *P* < 0.001), 15% within the 90-day follow-up period (HR = 0.85, 95%CI: 0.78–0.93, *P* < 0.001), and 12% within the 1-year follow-up period (HR = 0.88, 95%CI: 0.82–0.95, *P* = 0.001). As shown in Supplementary Table S2, model III was the best-fit model in the analysis of 30-day, 90-day and 1-year mortality, with the lowest AIC and BIC values. After PSM, the statin group also had a markedly lower incidence of all-cause mortality rate, and the risk of mortality reduced by 23% for the 30-day mortality (HR = 0.77, 95%CI: 0.68–0.88, *p* < 0.001), 16% for the 90-day mortality (HR = 0.84, 95%CI: 0.75–0.93, *p* < 0.001), and 12% for the 1-year mortality (HR = 0.88, 95%CI: 0.81–0.97, *p* = 0.007).

**TABLE 2:** Association between the statin group and all-cause mortality.

Mortality	30-day mortality		90-day mortality		1-year mortality	
	HR (95% CI)	*P*-value	HR (95% CI)	*P*-value	HR (95% CI)	*P*-value
Model I	0.54 (0.49–0.59)	<0.001	0.60 (0.56–0.65)	<0.001	0.67 (0.63–0.72)	<0.001
Model II	0.50 (0.45–0.55)	<0.001	0.58 (0.53–0.63)	<0.001	0.64 (0.60–0.69)	<0.001
Model III	0.82 (0.74–0.92)	<0.001	0.85 (0.78–0.93)	<0.001	0.88 (0.82–0.95)	0.001

HR, hazard ratio; CI, confidence interval.

### 3.4 Types and doses of statins and their relationship with all-cause mortality

The association of different types and doses of statins with all-cause mortality rate was evaluated in the original cohort. We excluded patients who received multiple types or doses of statins during the ICU period. Finally, 10,095 patients with a single dose record were retained.

The types of statins included atorvastatin (3,761 patients), rosuvastatin (470 patients), pravastatin (539 patients), simvastatin (1,486 patients) and lovastatin (19 patients). The results of multivariable COX regression analysis for different statin use are shown in Table 3. After controlling for all the underlying cofounders, we found that the 30-day all-cause mortality was remarkably lower in patients who were treated with rosuvastatin, simvastatin, or pravastatin. Specifically, the mortality risk reduced by 34% in patients who were treated with rosuvastatin (HR = 0.66, 95%CI: 0.49–0.89, *P* = 0.007), 20% in patients who were treated with simvastatin (HR = 0.80, 95%CI: 0.67–0.94, *P* = 0.008), and 22% in patients treated with pravastatin (HR = 0.78, 95%CI: 0.61–0.99, *P* = 0.0497) in comparison with the non-statin group. The 90-day all-cause mortality rate was also significantly lower in patients who were treated with atorvastatin, rosuvastatin or simvastatin (*P* = 0.049, *P* = 0.013, *P* = 0.011, respectively). Within the 1-year follow-up period, a 10% and 19% lower risk of death was observed in patients treated with atorvastatin (HR = 0.90, 95% CI: 0.82–0.98, *p* = 0.016) and rosuvastatin (HR = 0.81, 95%CI: 0.67–0.97, *P* = 0.023), respectively. There were no distinguishing differences in the all-cause mortality rates in patients who were treated with lovastatin whose sample size was too small, and the credibility intervals were wide. According to the HRs with 95%CIs, patients treated with rosuvastatin had the greatest reduction of mortality rate, and the risk of mortality rate reduced by 34%, 26%, and 19% in the 30-day, 90-day, and 1-year follow-up periods, respectively, in the adjusted model.

**TABLE 3:** The association between different statins and all-cause mortality.

Mortality	30-day mortality		90-day mortality		1-year mortality	
	HR (95% CI)	*P*-value	HR (95% CI)	*P*-value	HR (95% CI)	*P*-value
Non-statin	Reference					
Types of statins						
Atorvastatin	0.91 (0.81–1.03)	0.136	0.90 (0.81–0.99)	0.049	0.90 (0.82–0.98)	0.016
Rosuvastatin	0.66 (0.49–0.89)	0.007	0.74 (0.59–0.94)	0.013	0.81 (0.67–0.97)	0.023
Simvastatin	0.80 (0.67–0.94)	0.008	0.84 (0.74–0.96)	0.011	0.90 (0.81–1.00)	0.053
Pravastatin	0.78 (0.61–0.99)	0.0497	0.86 (0.71–1.05)	0.131	0.91 (0.78–1.07)	0.243
Lovastatin	0.00 (0.00 - Inf)	0.978	0.38 (0.09–1.52)	0.170	0.60 (0.25–1.45)	0.253
Doses of statins						
Low dose	0.81 (0.71–0.91)	<0.001	0.84 (0.76–0.92)	<0.001	0.87 (0.80–0.94)	<0.001
High dose	0.92 (0.80–1.06)	0.270	0.93 (0.83–1.05)	0.244	0.95 (0.86–1.04)	0.265

HR, hazard ratio; CI, confidence interval.

To further explore the impact of different doses of statins on the mortality rate of HF patients, statin use was divided into low-dose statin and high-dose statin groups. High-dose statin was defined as atorvastatin 80 mg, simvastatin 80 mg, pravastatin 40 mg, and rosuvastatin 20 mg per day (Pandit et al., 2016). 3,539 patients received high-dose statins and 2,736 patients received low-dose statins. As shown in Table 3, after controlling for all the underlying cofounders, the 30-day, 90-day and 1-year all-cause mortality rates were remarkably lower in patients receiving low-dose statins. Specifically, the risk of 30-day mortality reduced by 19% (HR = 0.81, 95%CI: 0.71–0.91, *P* < 0.001); the risk of 90-day mortality reduced by 16% (HR = 0.84, 95%CI: 0.76–0.92, *P* < 0.001); the risk of 1-year mortality reduced by 13% (HR = 0.87, 95%CI: 0.80–0.94, *P* < 0.001). The differences in the mortality rates between the high-dose statin group and the non-statin group were not significant. The risks of 30-day (HR = 0.87, 95%CI: 0.76–1.00, *P* = 0.059), 90-day (HR = 0.90, 95%CI: 0.80–1.00, *P* = 0.060), and 1-year mortality (HR = 0.92, 95%CI: 0.84–1.01, *P* = 0.073) were lower but not significant in the low-dose statin group campared with the high-dose statin group.

### 3.5 Subgroup analysis

We performed the subgroup analysis to explore the relationship between statin use and the 1-year mortality rate. The results are shown in Supplementary Figure S1 and suggest no interaction between stratified variables and statin exposure (P for interaction >0.05) except for hyperlipidemia and stroke (P for interaction <0.001). A remarkable link between statin use and the 1-year mortality rate was found in patients with hyperlipidemia and with or without stroke, while no significant correlation was observed in patients without hyperlipidemia.

## 4 Discussion

HF is a complex disease that is caused by structural and/or functional cardiac abnormality, leading to decreased cardiac output and/or increased intracardiac pressure (Ponikowski et al., 2016). Pharmacological treatment of HF includes diuretics, renin-angiotensin system inhibition with ACEI, ARB or ARNI, MRAs, sodium-glucose cotransporter inhibitors, beta-blockers, hydralazine and isosorbide dinitrate (Writing Committee and Members, 2022). Statins have a vital therapeutic role in patients with cardiovascular atherosclerosis (Delahoy et al., 2009) and are among the most widely prescribed medications for cardiovascular disease (CVDs) (Adhyaru and Jacobson, 2018). Statins were recommended for patients who have MI or ACS in their recent or remote past to prevent symptomatic HF and adverse cardiovascular events (Writing Committee and Members, 2022). However, conflicting evidence exists regarding the benefit of statins in HF patients.

According to experimental evidence, HF patients may benefit from statins, which, beyond increasing the hepatic uptake of cholesterol and lowering lipids in the blood (Stancu and Sima, 2001), have several pleiotropic actions. Evidence suggests that statins interact with the nitric oxide (NO) pathway and increase NO bioavailability to improve endothelial function in a cholesterol-independent way (Tousoulis et al., 2007). Moreover, statins have anti-inflammatory, immunomodulatory and antioxidant effects (Blanco-Colio et al., 2003; Davignon et al., 2004). Based on experimental evidence, statins may inhibit fibrotic and hypertrophic remodeling in the heart, and they can affect the expression and function of inflammatory cytokines and signaling molecules involved in cardiac remodeling (Gorabi et al., 2021). Nevertheless, there are potential harmful effects of statins. Statins decrease the circulating cholesterol, and low TC was reported to be strongly related to the increased mortality rate in people with nonischemic, systolic HF (Afsarmanesh et al., 2006). Statin also decreased circulating coenzyme Q10 (CoQ10) (Qu et al., 2018), which has antioxidative activities. Some studies have indicated that the level of plasma CoQ10 was an independent predictor of mortality rate in congestive heart failure (CHF) patients and the CoQ10 deficiency might negatively affect the long-term prognosis in people with CHF (Molyneux et al., 2008). In addition, some studies have shown the effects of statins on vitamin D metabolism (Mazidi et al., 2017; Yavuz et al., 2009), and low vitamin D status was associated with the risk of ventricular remodeling and mortality in patients with HF (Brinkley et al., 2017).

There was debate regarding the efficacy of statin treatment in lowering the mortality of HF patients (Tavazzi et al., 2008; Kjekshus et al., 2007; Foody et al., 2006; Kaur et al., 2023). We examined whether using statins in the ICU has an association with the clinical outcome of people with HF. According to the baseline characteristics, patients who received statins in our cohort study seemed to be older, heavier and had higher incidence of co-morbidities such as diabetes, myocardial infarction, PVD, stroke, hyperlipidemia and CKD. Yet, the mortality rate was lower. After the potential confounding factors were adjusted using Cox regression models, both the short-term and long-term mortality rates, including the 30-day, 90-day and 1-year mortality, decreased in the statin group in comparison with the non-statin use group. This finding was also confirmed by the results of robustness tests. Our results were consistent with some previous research. A recent study conducted by Marume et al. (2019) revealed that all-cause mortality, non-cardiac death and rehospitalization of patients with HFpEF were all lower in the statin group. A nationwide prospective study indicated that in the propensity score matched population of 21,864 patients with HFrEF, statin-treated patients had an 83% 1-year survival compared to 79% for untreated patients (HR 0.81; 95% CI 0.76–0.86; *p* < 0.001) (Alehagen et al., 2015). Anderson et al. (2023) also found that statin use in the real-world management of HFrEF patients with atherosclerotic cardiovascular disease (ASCVD) or at high ASCVD risk was associated with a lower risk of major adverse cardiovascular events (e.g., death, myocardial infarction, stroke). In addition, some meta-analyses have also been designed to explore the effects of statins on mortality and suggested the benefits of statins in both HFpEF and HFrEF (Bielecka-Dabrowa et al., 2019; Kaur et al., 2023; Liu et al., 2014). In a real-world study, 960 HF patients with preserved or depressed left ventricular ejection fraction (LVEF) regardless of HF etiology were included and followed up for a maximum of 9.1 years, and statins were found to be independently and remarkably related to lower mortality risk (Gastelurrutia et al., 2012). However, two large randomized placebo-controlled trials, GISSI-HF and CORONA, showed no benefits of statin treatment in individuals with HF. In the GISSI-HF trial, 4,574 people with HF irrespective of cause and LVEF were recruited, and no remarkable differences in all-cause mortality as well as hospitalization were found for cardiovascular causes in the rosuvastatin group (Tavazzi et al., 2008). One of the main limitations of the GISSI-HF trial was that a third of the patients did not fully comply with the treatment assignment. In the CORONA trial, 5,011 people with ischemic HFrEF were included, and after a median follow-up period of 32.8 months, rosuvastatin did not lower the all-cause mortality or the primary end point (Kjekshus et al., 2007). Nonetheless, the trial had a study population with a mean age of 73 years and with advanced HF stages, and therefore, concerns exist for the generalizability of the trial results to the whole HF population (Techorueangwiwat et al., 2021). According to our subgroup analysis, hyperlipidemia and stroke interacted with statin exposure significantly, and no significant correlation between statins and the 1-year mortality rate was observed in people without hyperlipidemia. This may indicate that statins have a greater effect on reducing the 1-year all-cause mortality rate in HF patients combined with hyperlipidemia.

According to the available data, rosuvastatin has been shown to reduce low density lipoprotein (LDL) cholesterol more effectively (Wander et al., 2018; Olsson et al., 2002; Zhang et al., 2020) and have the greatest preventive effects for CVDs compared with other statins (Liu et al., 2023). In adults who have coronary artery disease, rosuvastatin and atorvastatin demonstrated comparable efficacy for the composite end point of all-cause death (Lee et al., 2023). However, in HF patients, lipophilic statins (e.g., atorvastatin, and simvastatin) seem to be much more favorable compared with hydrophilic statins (e.g., rosuvastatin, and pravastatin) (Bielecka-Dabrowa et al., 2019; El et al., 2021), and this might be explained by the fact that lipophilic statins have a better ability to penetrate cardiac muscle cells and may better influence the myocardium through pleiotropic effects (Techorueangwiwat et al., 2021). In our cohort study, both lipophilic statins and hydrophilic statins were found to be related to lower short-term as well as long-term all-cause mortality rates. Among different types of statins, rosuvastatin was associated with the greatest reduction of the 30-day, 90-day and 1-year mortality rates. The main reason for the inconsistency of our results with previous research may lie in the different study populations. To the best of our knowledge, the current research is the first to compare the impact of different statins on mortality in the ICU population.

High-dose statins can further reduce the risk of atherosclerotic cardiovascular disease (ASCVD) events (Barter, 2018) and are recommended for patients with acute coronary syndrome (Anayat et al., 2023). Nonetheless, the safety of high-dose statins has long been concerned. A higher dose of statins is associated with increased risks of myopathy and elevated transaminases compared to a lower dose of statins (Josan and McAlister, 2007). It is proposed that short-term use of low-dose statins may be a promising option for preventing and treating acute sarcopenia (De Spiegeleer et al., 2023). However, the impact of different doses of statin on HF is not clear. Our results showed that a lower dose of statins was associated with lower short-term and long-term mortality rates in HF patients, while a higher dose did not, even though there was no difference between higher and lower doses. The results might be explained by the hypothesis that a high dose of statins leads to disease progress in HF by inhibiting CoQ10 synthesis and intensifying hypertrophy (Niazi et al., 2020). Future randomized trials with longer follow-up periods and larger sample sizes are needed to determine whether differences exist in the efficacy and safety of different types and doses of statins.

Moreover, statins are also beneficial for patients with higher inflammatory states such as frail older adults, critically ill patients or patients with infections (De Spiegeleer et al., 2022; Chen et al., 2023; Navia et al., 2022; Xu et al., 2022). However, these issues are not analyzed in our research. Clinical trials with stricter inclusion criteria and indicators such as frailty index and inflammation index are imperative to eliminate the influence of other factors.

There were several limitations in this study. First, our study is a single-center retrospective observational study that has a small sample size. Future prospective multi-center randomized controlled trials are needed to obtain more information. Second, our study did not include all potential confounding variables, and therefore it may not be sufficient to exclude the influence of all confounding variables. Third, we did not stratify the patients according to the left ventricle ejection fraction because of missing values. Finally, we were unable to obtain the medication information after hospitalization. Therefore, we were not sure of whether the patients continued statins after being discharged from the ICU, and this may have some impacts on the results.

## 5 Conclusion

In conclusion, the current retrospective research of a large database showed that statin use was linked to reduced short-term and long-term all-cause mortality rates in critically ill people with HF. Rosuvastatin was associated with the greatest reduction in all-cause mortality rate. Low-dose statins can significantly reduce short-term and long-term mortality rates, while high-dose statins are not significantly correlated with mortality. However, the results are not conclusive and should be interpreted with caution.

## Data Availability

The original contributions presented in the study are included in the article/Supplementary Material, further inquiries can be directed to the corresponding author.
