# Supplementary material for: The impact of statin use on short-term and long-term mortality in patients with heart failure
Source: Front Pharmacol. 2024 Sep 26;15:1397763. doi: 10.3389/fphar.2024.1397763 (PMC11464369; doi:10.3389/fphar.2024.1397763)

**Table S1.** Mortality rates in patients with heart failure in the statin group and non-statin groups

| Mortality,N(%) | Original cohort | | | PSM cohort | | |
| --- | --- | --- | --- | --- | --- | --- |
|  | Statin | Non-statin | P value | Statin | Non-statin | P value |
| 30 days | 1056(14.0%) | 915(24.0%) | <0.001 | 414(17.0%) | 520(21.3%) | <0.001 |
| 90 days | 1617(21.4%) | 1238(32.4%) | <0.001 | 631(25.9%) | 723(29.6%) | <0.001 |
| 1 year | 2440(32.3%) | 1634(42.8%) | <0.001 | 893(36.6%) | 973(39.9%) | 0.007 |

**Table S2.** Assessment of the goodness-of-fit of models.

|  | 30-day mortality | | 90-day mortality | | 1-year mortality | |
| --- | --- | --- | --- | --- | --- | --- |
|  | AIC | BIC | AIC | BIC | AIC | BIC |
| Model I | 36276.41 | 36282.00 | 52371.91 | 52377.87 | 74282.24 | 74288.55 |
| Model II | 34840.32 | 34974.39 | 50653.22 | 50798.18 | 72291.65 | 72443.15 |
| Model III | 33096.08 | 33403.32 | 48940.81 | 49268.43 | 70185.26 | 70532.44 |

**Figure S1.** The association between statin administration and 1-year all-cause mortality in subgroups.


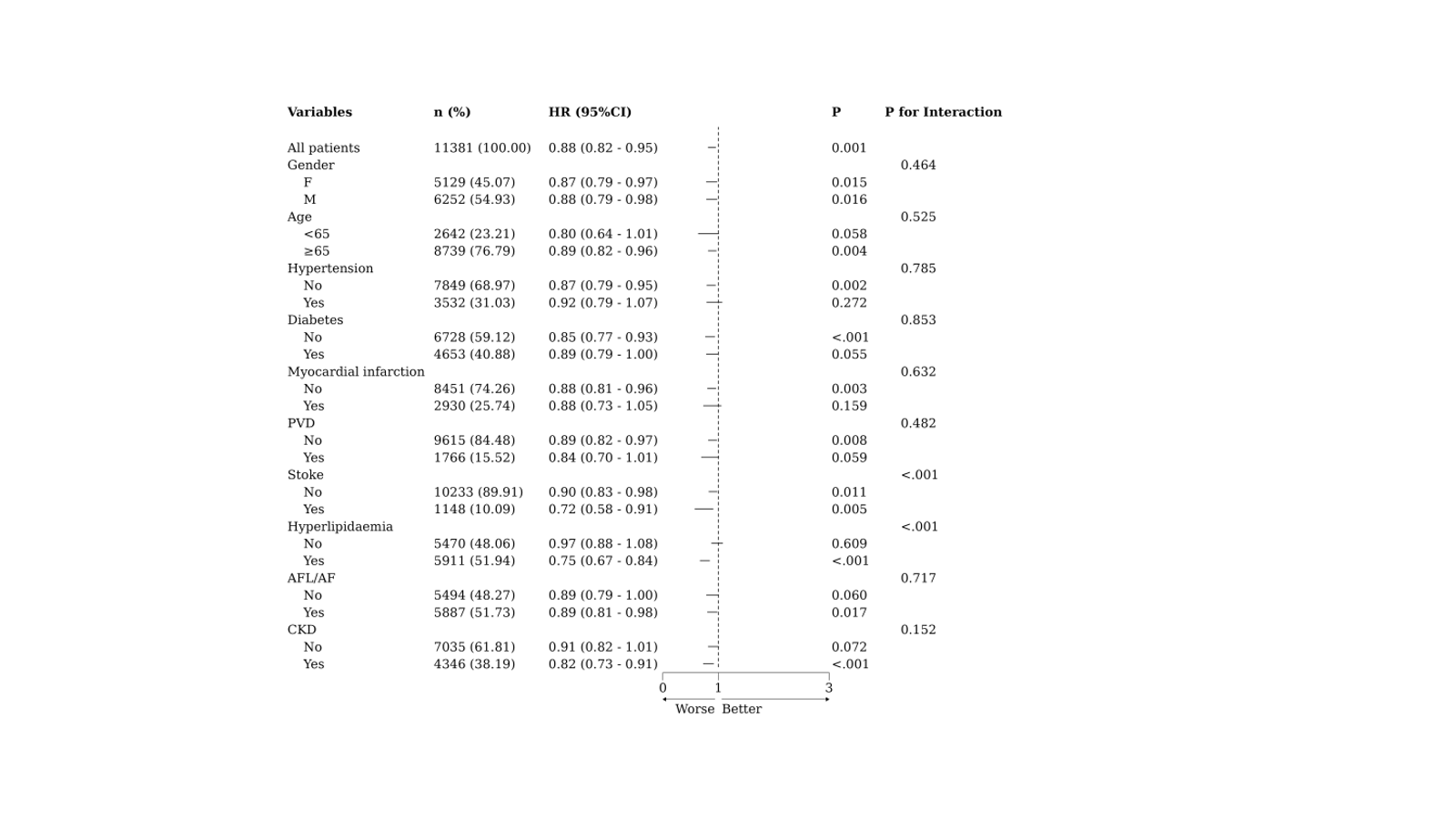

Supplement: Supplementary file 1 [file DataSheet1.docx]
